# Assessment of the Anti-Acne Properties of Some Medicinal Plants and Development of an Herbal Anti-Acne Formulation

**DOI:** 10.3390/pharmaceutics17030317

**Published:** 2025-03-01

**Authors:** F. Sezer Senol Deniz, Ozlem Oyardı, Cagla Bozkurt Guzel, Tahir Emre Yalcın, Serkan Yiğitkan, Yuksel Kan, Nurver Ulger Toprak, Ilkay Erdogan Orhan

**Affiliations:** 1 Department of Pharmacognosy, Faculty of Pharmacy, Gazi University, 06330 Ankara, Türkiye; 2 Department of Pharmaceutical Microbiology, Faculty of Pharmacy, Gazi University, 06330 Ankara, Türkiye; ozlemoyardi@gazi.edu.tr; 3 Department of Pharmaceutical Microbiology, Faculty of Pharmacy, Istanbul University, 34116 Istanbul, Türkiye; cagla.bozkurt@istanbul.edu.tr; 4 Department of Pharmaceutical Technology, Faculty of Pharmacy, Gazi University, 06330 Ankara, Türkiye; emreyalcin@gazi.edu.tr; 5 Department of Pharmacognosy, Faculty of Pharmacy, Dicle University, 21280 Diyarbakır, Türkiye; serkan.yigitkan@dicle.edu.tr; 6 Department of Medicinal Plants, Agriculture Faculty, Selcuk University, 42130 Konya, Türkiye; ykan@selcuk.edu.tr; 7 Department of Medical Microbiology, Basic Medical Sciences, School of Medicine, Marmara University, 34854 Istanbul, Türkiye; nulger@marmara.edu.tr; 8 Department of Pharmacognosy, Faculty of Pharmacy, Lokman Hekim University, 06510 Ankara, Türkiye; ilkay.erdoganorhan@lokmanhekim.edu.tr; 9 Turkish Academy of Sciences (TÜBA), Vedat Dalokay Cad., No. 112, 06670 Ankara, Türkiye

**Keywords:** acne, *Cutibacterium acnes*, LC-MS/MS, medicinal plant, formulation, nanoemulsion

## Abstract

**Background**: Acne is a prevalent dermatological condition characterized by the blockage of hair follicles and sebaceous glands, leading to the formation of acne. The anaerobe pathogen *Cutibacterium acnes* (formerly known as *Propionibacterium acnes*) plays an essential role in the pathogenesis of acne, for which generally antimicrobial treatment is required. Acne is a substantial health concern, and continuing research is being conducted to discover novel and efficacious remedies. The antimicrobial activity of plants has been demonstrated in numerous studies, and they are still targeted organisms in drug development. Studies showing that plants are effective against acne pathogens have also been reported. **Methods**: The antimicrobial activity of the hydroethanolic extracts prepared from 30 plant species was determined against *C. acnes* standard strains (*C. acnes* Scholz and Kilian ATCC 11827 and ATCC 11828) and 30 clinical isolates in our preliminary screening. Since acne is an inflammatory skin disease, the anti-inflammatory effect of six active extracts against *C. acnes* was determined through the in vitro inhibition of collagenase, lipoxygenase (LOX), hyaluronidase and xanthine oxidase (XO) enzymes. **Results**: *Cotinus coggygria* Scop. leaf extract displayed the highest hyaluronidase and collagenase inhibition (79.75% and 52.52%, respectively), while the extract from the aerial parts of *Helichrysum arenarium* (L.) Moench demonstrated a potent XO inhibitory effect (82.51%). Therefore, these two extracts have been chosen for further studies, and LC/MS-MS was used to determine the phenolic profiles of these extracts. **Conclusions**: Subsequently, nanoemulgels were formulated with the active extracts to develop a prototype herbal anti-acne product, and characterization studies of the formulations were conducted.

## 1. Introduction

Acne is an inflammatory skin disease that can affect human health and quality of life psychologically and socially. In particular, moderate or severe acne has dramatic effects on quality of life, such as decreased self-confidence and a deterioration of social relations, by affecting one’s physical appearance [1,2,3]. Numerous studies conducted in different countries have shown that acne is prevalent worldwide, especially among teenagers and young adults [4]. Although factors such as hormonal activity, stress, and genetic predisposition are prevalent in the etiology of acne, the most common cause is microbial colonization caused by *Cutibacterium acnes* and inflammation at the site of infection. The topical and systemic use of antibiotics that are effective for *C. acnes* is essential in treating acne. However, one of the biggest problems affecting the treatment of acne with antibiotics is the development of resistance to *C. acnes*, especially in monotherapy [5]. In addition to being naturally resistant to 5-nitroimidazole agents (metronidazole, tinidazole and ornidazole), aminoglycosides, sulfonamides and mupirocine, it shows a high level of resistance to erythromycin and clindamycin and a low level of resistance to tetracycline, which are among the most commonly used antibiotics topically in the treatment of acne [6]. To prevent the increasing problem of resistance, it is necessary to limit the use of antibiotics, develop new treatment methods, and, if required, apply combined treatment with antibiotics. For this purpose, using effective herbal extracts on *C. acnes* may prevent side effects related to antibiotics, slow the development of resistance, and develop an additional treatment strategy. The extracts prepared from 30 plants, which were chosen due to their application in traditional medicine in various countries against skin diseases and/or their abundant essential oil content and significant antimicrobial effects, were tested in our pre-screening against *C. acnes*.

Lipoxygenases (LOX) are members of the non-heme-iron-containing dioxygenase family, distributed in animals and plants. In mammalian cells, these enzymes are involved in the biosynthesis of various bioregulatory compounds such as hydroxyeicosatetraenoic acids, leukotrienes, lipoxins and hepoxylin. These compounds also contribute to various ailments, such as bronchial asthma and inflammation. Tissue inflammation is also an important factor in the formation of acne. It has been observed that 5-LOX activity increases in acne-prone skin compared to healthy skin. LOX products play a role in inflammatory skin diseases characterized by the hyperproliferation of keratinocytes. Therefore, LOX inhibition has become an essential target for reducing inflammatory processes in the sebaceous gland [7,8,9]. Xanthine oxidase (XO) is a purine catabolism enzyme that catalyzes the conversion of hypoxanthine to xanthine and xanthine to uric acid. In normal tissues, XO exists primarily as xanthine dehydrogenase (XD), which does not produce free radicals. During inflammatory processes such as acne, XD is transformed into XO, the biological source of free oxygen radicals. The decrease in the body’s antioxidant defense system also plays a vital role in the pathogenesis of acne. Studies have shown that the serum malondialdehyde content and XO activity are high, while the serum catalase and superoxide dismutase activity are low in patients with acne [10,11,12,13]. UV exposure also increases the activity of matrix metalloproteinases (MMPs) by causing the formation of reactive oxygen species. Studies have shown that MMPs such as collagenase are induced in inflamed skin, such as photo-aged skin [14]. Therefore, MMPs are attractive targets in the search for new anticancer, antiarthritis and other beneficial pharmacological agents that regulate anti-inflammatory processes [15,16]. Hyaluronic acid (HA), or hyaluronan, is commonly found in the extracellular matrix of soft connective tissues such as the umbilical cord, skin and synovial fluid. HA synthesis and breakdown rates in connective tissue change over time. In inflammatory conditions, an imbalance is observed caused by increased fragmentation or decreased synthesis. These imbalances can be attributed to increased oxidative stress, increased inflammatory mediator formation, decreased HA production, and increased breakdown of HA. Increased hyaluronidase activity contributes to degenerative changes in connective tissue. Thus, hyaluronidase inhibitors are potent regulators that maintain HA homeostasis and can be used as anti-inflammatory, anti-aging, antimicrobial, anticancer, anti-venom/toxin and contraceptive agents [17,18,19].

The transdermal drug delivery system (TDDS) has long been utilized to administer various pharmacological agents through the skin. Nanoemulgel, regarded as a promising TDDS, is a hybrid formulation in which nanoemulsions (NEs) are incorporated into a hydrogel matrix to improve therapeutic efficacy and application properties. These systems are thermodynamically stable dispersions with a droplet size of 20–500 nm [20] and can effectively deliver active ingredients to targeted areas in the skin. Lately, there has been a notable surge in interest in utilizing NEs for acne treatment. Due to their small droplet sizes, NEs can easily penetrate the skin and may be used to reduce inflammation in acne treatment. It has been indicated in the literature that nanocarriers smaller than 300 nm may penetrate the skin more efficiently [21]. The present study aims to develop a novel nanoemulgel formulation combining NEs (<300 nm) containing extracts with hydrogels for acne treatment.

## 2. Materials and Methods

### 2.1. Collection and Extraction of Plant Samples

Thirty plants used in the screening were freshly collected and identified by Prof. Dr. Yüksel Kan from the Faculty of Agriculture, Selcuk University (Konya, Türkiye) over a three-year period. The herbarium specimens were deposited at Selcuk University Medicinal and Endemic Plants Herbarium (SÜTEB-Medicinal and Endemic Plants Education and Research Farm, Selcuk University, Konya, Türkiye). The plant materials were ground to fine powder after being dried in the shade. The powdered plants were then accurately weighed (15 g), left to macerate for 1 week at room temperature after adding ethanol (80%, 150 mL) and occasionally shaken by hand. At the end of the period, the filtered hydroethanolic parts were condensed in the evaporator until dry using a 40 °C water bath under low pressure. The obtained extracts were weighed, and their yields were calculated and stored at +4 °C for experimental use (Table 1).

### 2.2. Determination of Antimicrobial Activity

#### 2.2.1. Bacterial Strains Used and Culture Conditions

The antimicrobial activity of the prepared hydroethanolic extracts against the standard strains of *Cutibacterium acnes* (*C. acnes* Scholz and Kilian ATCC 11827 and ATCC 11828) was determined. Afterwards, a total of 6 extracts prepared from *Cotinus coggygria*, *Helichrysum arenarium*, *Origanum vulgare*, *Pistacia vera*, *Salvia fruticosa* and *Sideritis congesta*, which were effective against standard strains, were screened for their antimicrobial effects against clinical strains, and their minimum inhibitory concentrations (MICs) as MIC_50_ and MIC_90_ values were calculated. A total of 30 clinical strains of *C. acnes* were obtained from the cultural collection of the Department of Medical Microbiology, Faculty of Medicine, Marmara University (Istanbul, Türkiye). Among them, 19 strains were isolated from the skin of patients with active acne, while 11 were isolated from the skin of healthy people. *C. acnes* ATCC 11827 and 11828 were employed as control strains.

The bacteria were frozen in 60% Brucella broth/40% glycerol and then stored at −80 °C. Enriched Brucella agar (Brucella agar, 5% sheep blood, hemin and vitamin K) was used to culture the bacteria. The bacteria were then grown in an anaerobic environment created using anaerobic jars and anaerobic generation kits.

#### 2.2.2. Broth Microdilution Test

The MIC values of the prepared extracts were determined using the broth microdilution method. One to two similar colonies of bacteria were taken from enriched brucella agar, suspended in brain heart infusion broth (BHIB) and adjusted to 0.5 McFarland standard using a McFarland Density Meter (Biosan) device, which is equal to 108 colony-forming units (cfu)/mL. The bacterial inoculum was diluted to 106 cfu/mL in infusion broth for the experiment. The number of viable cells was also determined in each study.

Two-fold diluted extracts with concentrations ranging from 5000 to 2.44 μg/mL (5000, 2500, 1250, 625, 312.5, 156.25, 78.12, 39.06, 19.53, 9.76, 4.88 and 2.44 μg/mL) were prepared in a 96-well microplate. The prepared bacterial inoculum was added to the microplate wells with a final concentration of 5 × 10^5^ in the wells. The lid of the microplate was closed, placed in a plastic bag to prevent evaporation and incubated in an anaerobic environment at 37 °C for at least 48 h. As a result of incubation, the lowest concentration at which bacterial growth was not observed was accepted as the MIC value. The experiments were repeated 3 times and conducted following the standards of the Clinical and Laboratory Standards Institute (CLSI) [22].

### 2.3. In Vitro Enzyme Inhibition Tests

#### 2.3.1. Hyaluronidase Inhibition

Hyaluronidase inhibition was measured using the spectrophotometric method applied by Tu and Tawata [23] with minor modifications [24]. According to the method protocol, 5 μL of type 1-S bovine hyaluronidase (Sigma-Aldrich, St. Louis, MO, USA, EC 3.2.1.35) was mixed with 25 μL of sample solution, and pre-incubation was applied at 37 °C for 10 min. A volume of 10 μL of phosphate buffer (300 mM) at pH of 5.35 was added to the mixture, which was then left to incubate at 37 °C for 10 min. Then, 10 μL of hyaluronic acid (0.03%) was added, and the mixture was incubated for 45 min. Finally, 100 μL of acidic albumin solution was added to the mixture and incubated at room temperature for 10 min. After incubation, the absorbance was measured at 600 nm in an ELISA microplate reader (Molecular Devices, Spectramax ABS Plus microplate reader, Sunnyvale, CA, USA). Tannic acid was used as a reference. Hyaluronidase inhibitions (%) by the samples were calculated using the formula below. Each sample was studied in 3 parallels, and the results are given as the mean of the % inhibitions obtained from 3 experiments ± standard deviation (S.D.).

Inhibition % = 100 − [(A_1_/A_2_) × 100]

A_1_ = Absorbance of sample solutions at a wavelength of 600 nm; A_2_ = Average absorbance of control solutions at a wavelength of 600 nm.

#### 2.3.2. Collagenase Inhibition

Collagenase inhibition was measured using the modified spectrophotometric method developed by Van Wart and Steinbrink [25,26]. As a source of enzymes, *Clostridium histolyticum* collagenase (Sigma-Aldrich, St. Louis, MO, USA, EC 3.4.23.3) was used, and as the substrate, N-(3-[2-furyl]acryloyl)-Leu-Gly-Pro-Ala (FALGPA) (Sigma-Aldrich, St. Louis, MO, USA) was used. A total of 0.8 units/mL of the enzyme was stored in 50 mM tricine buffer (10 mM CaCl_2_ at a pH of 7.5) with 400 mM NaCl. The substrate is dissolved in the same buffer with FALGPA (2 mM). Volumes of 25 μL of buffer, 25 μL of DMSO or extract, and 25 μL of enzyme were mixed in a 96-well microplate. After 15 min of pre-incubation, 50 μL of substrate was added. Absorbance at 340 nm for 20 min with an interval of 2 min was recorded on an ELISA microplate reader (Molecular Devices, Spectramax ABS Plus microplate reader, Sunnyvale, CA, USA). Reference 1,10-phenanthroline (Sigma-Aldrich, St. Louis, MO, USA) was used as the positive control, and DMSO was used as the negative control. Collagenase inhibitions (%) by the extracts were calculated using the formula below. Each sample was studied in 3 parallels, and the results are given as the mean of the % inhibitions obtained from 3 experiments ± S.D.

Inhibition % = 100 − [(A_1_/A_2_) × 100]

A_1_ = Absorbance changes in sample solutions at a wavelength of 340 nm; A_2_ = Average absorbance changes in control solutions at a wavelength of 340 nm.

#### 2.3.3. XO Inhibition

XO inhibition method is based on spectrophotometric measurement of the absorbance of uric acid formed due to the enzyme reaction at 295 nm. Volumes of 150 μL 0.1 M phosphate buffer (pH 7.5), 10 μL of DMSO or sample (plant extract, reference inhibitor or negative control) and 20 μL of the enzyme (0.003 units/well) (Sigma-Aldrich, St. Louis, MO, EC 1.17.3.2) were mixed in a 96-well microplate. After 10 min of pre-incubation, 20 μL of xanthine (0.1 mM) was added as a substrate, and the reaction was initiated [27]. Absorbance change was measured in an ELISA microplate reader (Molecular Devices, Spectramax ABS Plus microplate reader, Sunnyvale, CA, USA) for 10 min. Allopurinol was used as a reference, and DMSO was used as a negative control. XO % inhibitions of the samples were calculated according to the formula below. Each sample was studied in 3 parallels, and the results were given as the mean of % inhibitions obtained from 3 experiments ± S.D.

Inhibition % = 100 − [(A_1_/A_2_) × 100]

A_1_ = Absorbance changes in sample solutions at a wavelength of 295 nm; A_2_ = Average absorbance changes in control solutions at a wavelength of 295 nm.

#### 2.3.4. LOX Inhibition

LOX inhibitory capacity of the extracts was tested according to the method of Chung et al. [28] with slight modifications. In a 96-well microplate, 10 µL sample (plant extract, reference inhibitor or negative control) and 60 µL buffer (50 mM Tris HCl buffer, pH 7.4) followed by 20 µL LOX from *Glycine max* (Sigma-Aldrich, St. Louis, MO, USA, EC 1.13.11.12) prepared in buffer (500 U/mL) were mixed and incubated at 25 °C for 5 min. The reaction was initiated by adding 10 µL linoleic acid (12.5 mM) dissolved in Tween 20 and Tris HCl buffer, and the reaction mixture was incubated at 25 °C for 20 min in the dark. The assay was terminated by the addition of 100 μL freshly prepared FOX reagent [sulfuric acid (3 M), xylenol orange (10 mM), ammonium iron (II) sulfate (10 mM), water: methanol (9:1)]. After termination, the Fe^3+^–dye complex was allowed to develop in the dark for 30 min at 25 °C before being measured at 560 nm on a microplate reader (Molecular Devices, Spectramax ABS Plus microplate reader, Sunnyvale, CA, USA). DMSO was used as the negative control, and baicalein (Sigma-Aldrich, St. Louis, MO, USA) was used as a reference inhibitor. Each sample was studied in 3 parallels, and the results are given as the mean of % inhibitions obtained from 3 experiments ± S.D.

Inhibition % = 100 − [(A_1_/A_2_) × 100]

A_1_= Absorbance of sample solutions at a wavelength of 560 nm; A_2_= Average absorbance of control solutions at a wavelength of 560 nm.

### 2.4. Statistical Analyses

The data for the percentage of enzyme inhibitory effects were statistically examined using one-way ANOVA followed by Dunnett’s multiple comparison test to compare the positive control with the test groups (GraphPad Prism 6.01). The values of *p* ≤ 0.05 were considered statistically significant.

### 2.5. LC-MS/MS Analyses

LC-MS/MS analyses were conducted to determine the general phenolic profiles of *C. coggygria* and *H. arenarium* extracts. In the present study, these extracts were determined to have noteworthy antimicrobial activity and anti-inflammatory effects in vitro and were used to prepare formulations.

Stock solutions were prepared from the crude extracts at 4000 µg/mL concentrations, diluted to 1000 µg/mL and filtrated with a 0.2 μm syringe filter prior to LC-MS/MS analysis (Shimadzu 8040 model). Thirty phenolic and flavonoid-derivative compounds were determined in extracts using the LC-MS/MS method previously developed and validated by our research group. A Shimadzu–Nexera ultra-high-performance liquid chromatography (UHPLC) system, integrated with a tandem mass spectrometer, was employed for the quantitative assessment of 53 phytochemicals. The reversed-phase UHPLC was equipped with an autosampler (SIL-30AC model), a column oven (CTO-10ASvp model), binary pumps (LC-30AD model), and a degasser (DGU-20A3R model). The chromatographic conditions were optimized to achieve optimum separation for 53 phytochemicals and overcome the suppression effects. Various columns, including the Agilent Poroshell 120 EC-C18 (150 mm × 2.1 mm, 2.7 µm) and RP-C18 Inertsil ODS-4 (100 mm × 2.1 mm, 2 µm), along with diverse mobile phases (B) (such as acetonitrile and methanol), multiple mobile-phase additives (including ammonium formate, formic acid, ammonium acetate and acetic acid) and a range of column temperatures from 25 °C to 40 °C were systematically tested until optimal conditions were established. Consequently, the chromatographic separation was performed on a reversed-phase Agilent Poroshell 120 EC-C18 model (150 mm × 2.1 mm, 2.7 µm) analytical column. The column temperature was set to 40 °C. The elution gradient was composed of eluent A (water + 5 mM ammonium formate + 0.1% formic acid) and eluent B (methanol + 5 mM ammonium formate + 0.1% formic acid). The following gradient elution profile was used: 20–100% B (0–25 min), 100% B (25–35 min), and 20% B (35–45 min). Furthermore, the solvent flow rate and injection volume were settled as 0.5 mL/min and 5 µL, respectively.

The mass spectrometric detection was carried out using a Shimadzu LCMS-8040 model tandem mass spectrometer equipped with an electrospray ionization (ESI) source operating in both negative and positive ionization modes. LC-ESI-MS/MS data were acquired and processed by LabSolutions software (Shimadzu, Kyoto, Japan). The MRM (multiple reaction monitoring) mode was used for the quantification of the phytochemicals. The MRM method was optimized to selectively detect and quantify phytochemical compounds based on the screening of specified precursor phytochemical-to-fragment ion transitions. The collision energies (CEs) were optimized to generate optimal phytochemical fragmentation and maximal transmission of the desired product ions. The MS operating conditions were applied as follows: drying gas (N_2_) flow, 15 L/min; nebulizing gas (N_2_) flow, 3 L/min; DL temperature, 250 °C; heat block temperature, 400 °C; and interface temperature, 350 °C [29]. The limit of detection (LOD) was between 0.05 and 25.8 µg/L, while the limit of quantification (LOQ) was between 0.17 and 85.9 µg/L. The recoveries of phenolic compounds varied between 96.9 and 106.2%.

### 2.6. Preparation of Formulations

#### 2.6.1. Preparation of NEs

NEs were prepared according to the spontaneous emulsification technique [30,31]. The effects of varying amounts of medium-chain triglyceride (MCT, Lipoid GmbH, Germany), α-tocopherol and oleic acid (Lipoid GmbH, Ludwigshafen, Germany) on the accelerated stability and characterization of the formulations were investigated. The formulation codes and their contents are presented in Table 2. After determining the optimum amounts for these components for formulation development, NEs with the extracts were prepared. The required amounts of MCT, L-α-phosphatidylcholine (soy, PC, 95%, Avanti Polar Lipid Inc., Alabaster, AL, USA), α-tocopherol (Sigma-Aldrich, St. Louis, MO, USA) and oleic acid were dissolved in ethanol (30 mL, Isolab, Wertheim, Germany) to form the oil phase. The amount of PC (600 mg) was kept constant across all formulations. For extract-loaded NEs, the extracts (200 mg) were added to the oil phase. The aqueous phase (80 mL) was prepared by dissolving the required amount of polysorbate 80 (2% *w*/*v*, Isolab, Wertheim, Germany) and sodium benzoate (0.2% *w*/*v*) in purified water. Emulsification was carried out by gradually adding the oil phase to the aqueous phase while stirring with a magnetic stirrer at 500 rpm at room temperature. The mixture was evaporated in a round-bottom flask with a rotary evaporator at 43 °C until the final volume reached 20–30 mL.

#### 2.6.2. Accelerated Stability Tests

The developed formulations underwent accelerated stability evaluation through the following experiments:

Centrifugation test: The stability of the NEs and nanoemulgels (NEGs) was assessed using a centrifugation test, in which each formulation was subjected to 5000 rpm for 30 min across three cycles for NEs and one cycle for NEGs [32].

Freeze–thaw cycle: The NEs underwent three freeze–thaw cycles, with each cycle consisting of storage at −20 °C for at least 48 h, followed by storage at 25 °C for at least 48 h [33].

Heating–cooling cycle: The stability of the NEs was also evaluated using a heating–cooling test for three cycles, with each cycle involving storage at 4 °C for at least 48 h, followed by storage at 40 °C for at least 48 h [34]. Stability studies comprising centrifugation, heating–cooling cycle and freeze–thaw cycle were performed to evaluate the phase separation.

#### 2.6.3. Preparation of Chitosan/Hydroxypropyl Methylcellulose (CS/HPMC) Hydrogel

Initially, a chitosan (CS) solution was prepared prior to the formulation of the low-molecular-weight CS/hydroxypropyl methylcellulose (HPMC K15M, Colorcon, Dartford, UK) hydrogel. The detailed process for hydrogel preparation is as follows: CS powder (1% *w*/*v*) was dissolved in a 1% *v*/*v* aqueous glacial acetic acid solution at room temperature and then centrifuged (1500–3000 rpm for 10 min) to remove insoluble impurities [35]. Hydrogels were prepared by adding 1.6 g of HPMC K15M to 40 g 1% *w*/*v* chitosan solution. The mixture was stored in the refrigerator and occasionally stirred with a glass rod until a clear solution was obtained [36].

#### 2.6.4. Preparation of NEGs

Extract-loaded NEs were combined with CS/HPMC gelling solution at a 1:3 *w*/*w* ratio and under gentle stirring. The uniform semisolid NEGs were obtained.

### 2.7. Characterization of NEs and NEGs

#### 2.7.1. Droplet Size, Polydispersity Index and Zeta Potential

Malvern Zetasizer (Nano ZS, Malvern Instruments, Worcestershire, UK) was used to determine the size, zeta potential and polydispersity index (PDI) of NEs and NEGs [37]. Before measurements, the samples were dispersed in a 1 mM NaCl solution [38]. All measurements were carried out in triplicate (n = 3).

#### 2.7.2. Transmission Electron Microscopy (TEM)

Morphological analyses of NEs and NEGs were performed by TEM (FEI Tecnai G2 Spirit Bio-Twin CTEM instrument, Hillsboro, OR, USA). The sample-loaded copper grid was stained with 1% (*w*/*v*) sodium phosphotungstate to obtain more explicit images. After that, the samples were dried in room conditions before being analyzed.

## 3. Results

### 3.1. Antimicrobial Activity

The MIC (µg/mL) values of the extracts against the standard *C. acnes* ATCC 11828 strain are provided in Table 3. The MIC (µg/mL) values of the six extracts (*C. coggygria*, *H. arenarium*, *O. vulgare*, *P. vera*, *S. fruticose* and *S. congesta*) that were found effective against the *C. acnes* clinical strains are presented in Table 4, while the MIC_50_ and MIC_90_ values are given in Table 5. According to the MIC values, *H. arenarium* and *P. vera* extracts exhibited the highest potency against *C. acnes*.

### 3.2. In Vitro Enzyme Inhibition Tests

The findings regarding the inhibition of hyaluronidase, collagenase, XO and LOX enzymes by hydroethanolic extracts prepared from plant materials at a stock concentration of 2 mg/mL are presented in Table 6. The *C. coggygria* extract showed efficacy in inhibiting collagenase and hyaluronidase enzymes (52.52% and 79.75%, respectively), whereas both the *C. coggygria* (80.30%) and *H. arenarium* (82.51%) extracts exhibited potent inhibition in XO. Meanwhile, extracts of *O. vulgare* and *S. fruticosa* displayed an approximately 50% inhibition of LOX.

### 3.3. LC-MS/MS Analyses

**Figure 1:**
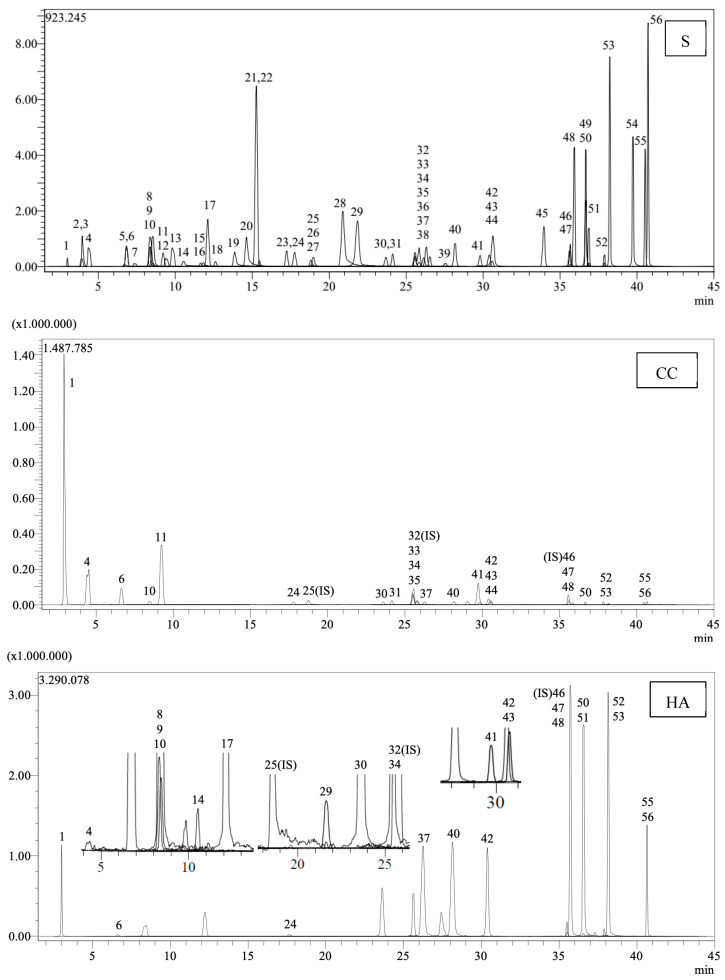
LC chromatograms of the standards (S) and extracts (CC: *Cotinus coggygria*, HA: *Helichrysum arenarium*) (1: Quinic acid. 2: Fumaric acid. 3: Aconitic acid. 4: Gallic acid. 5: Epigallocatechin. 6: Protocatechuic acid. 7: Catechin. 8: Gentisic acid. 9: Chlorogenic acid. 10: Protocatechuic aldehyde. 11: Tannic acid. 12: Epigallocatechingallate. 13: 1,5-dicaffeoylquinic acid. 14: 4-OH Benzoic acid. 15: Epicatechin. 16: Vanilic acid. 17: Caffeic acid. 18: Syringic acid. 19: Vanillin. 20: Syringic aldehyde. 21: Daidzin. 22: Epicatechingallate. 23: Piceid. 24: p-Coumaric acid. 26: Ferulic acid. 27: Sinapic acid. 28: Coumarin. 29: Salicylic acid. 30: Cynaroside. 31: Miquelianin. 33: Rutin. 34: Isoquercitrin. 35: Hesperidin. 36: o-Coumaric acid. 37: Genistin. 38: Rosmarinic acid. 39: Ellagic acid. 40: Cosmosiin. 41: Quercitrin. 42: Astragalin. 43: Nicotiflorin. 44: Fisetin. 45: Daidzein. 47: Quercetin. 48: Naringenin. 49: Hesperetin. 50: Luteolin. 51: Genistein. 52: Kaempferol. 53: Apigenin. 54: Amentoflavone. 55: Chrysin. 56: Acacetin).

Table 7 enumerates the detected phenolic compounds and their concentrations (mg/g extract) in *C. coggygria* leaf and *H. arenarium* extracts. In our MS library of 53 standard phenolic compounds, quinic acid was identified as the predominant phenolic compound in the extracts. Figure 1 presents the LC chromatograms for the standards and extracts.

### 3.4. Characterization of the Formulations

**Figure 2:**
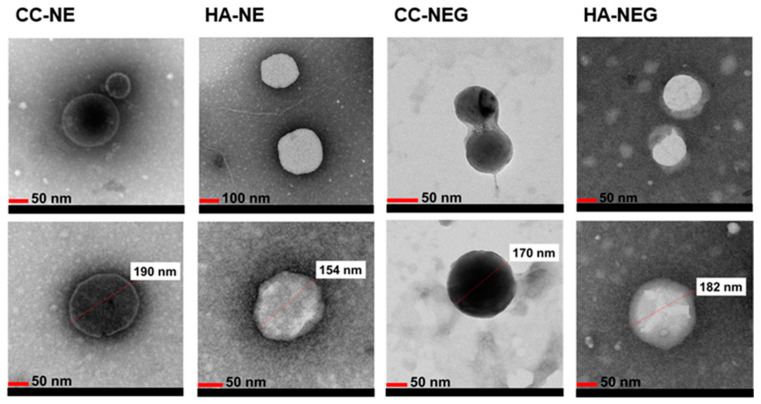
TEM images of extract-loaded NEs and NEGs (CC: *Cotinus coggygria*, HA: *Helichrysum arenarium*).

All the NEs tested to evaluate the effect of MCT successfully passed all accelerated stability tests with no phase separation. Similarly, no phase separation was observed in formulations containing α-tocopherol after the accelerated tests. The formulations loaded with the extracts also showed no phase separation during the stability tests. The droplet size, PDI and zeta potential of the formulated NEs, selected blank NEs and extract-loaded NEs are presented in Table 8 and Table 9. The TEM images of the extract-loaded NEs and NEGs are shown in Figure 2.

## 4. Discussion

*Acne vulgaris* is a shape-altering, long-term inflammatory disease of the pilosebaceous units. Acne is a chronic disease characterized by inflammatory and non-inflammatory lesions as a result of androgen-induced increased sebum production and altered keratinization, inflammation and bacterial colonization of *Cutibacterium acnes* [39,40,41]. Regarding the most common skin diseases, *acne vulgaris* ranks second in terms of its rate of incidence [42,43]. The psychological effects of acne include loss of self-confidence, depression, anxiety, and interpersonal and work-related difficulties. The clinical presentation of acne consists of black and white spots (comedones), papules, pustules, nodules and dimpled or hypertrophic scars, and the face, shoulders, upper chest and back may be affected [44,45]. The formation of *acne vulgaris* is mainly attributed to the increased production of androgens, which are present in men and women during puberty. Accordingly, the pilosebaceous units begin to produce more sebum, followed by follicular hyperkeratinization and the blockage of the hair follicles. Because of this, sebum cannot reach the skin’s surface, which encourages anaerobic bacteria, including *C. acnes*, to grow in the blocked follicle. These bacteria trigger an inflammatory response that manifests as an increased temperature, swelling, redness and pus on the skin [46]. Acne affects more than 85% of young people, and severe forms of the disease are prevalent in boys [47]. The global anti-acne cosmetics market is set at USD 5.29 billion in 2024 and is expected to increase to approximately USD 12.65 billion by 2034 [48]. Acne treatment aims to control existing lesions, prevent permanent scarring as much as possible, limit the duration of the disease, minimize morbidity and protect against the formation of new acne lesions. In addition to normalizing follicular keratinization and suppressing sebum production, anti-acne drugs can also act as an antibacterial against *C. acnes*, relieving inflammation and reducing sebum oxidation, which promotes the development of *C. acnes* through antioxidant action. Conventional acne treatment also includes topical products alone or, in severe cases, in combination with systemic treatments. Topically used agents include comedolytic agents, antibiotics and various anti-inflammatory drugs, while systemic agents include retinoids, antibiotics, zinc and hormones [49]. Topical treatment is standard for most patients with comedopapular acne; however, local and systemic treatments are needed for pustolocistic scarring acne. The disadvantages of conventional treatment include acne-causing bacteria (*C. acnes* and *S. epidermidis*), increasing antibiotic resistance, an increased incidence of pregnant women exposed to oral tretinoin, a known teratogen, and the poor safety profile of systemic retinoid therapy [50,51]. The use of herbal products in treating acne has increased due to their advantages, such as having better patient tolerance, a long history of use, fewer side effects and relatively better cost-effectiveness. Many plants with a history of use in traditional treatments have entered the growing cosmeceutical market as a result of research. The mechanisms of action of the plants used in the treatment of acne are due to their antibacterial activities against acne-causing bacteria and their effects on acne-related sebum activity, inflammation and hyperkeratinization.

In light of this information, the antimicrobial activities of the hydroethanolic extracts prepared from 30 medicinal plants against the standard strains of *C. acnes* were investigated in this study. As a result of the first screening of these extracts, the efficacy of six promising plant extracts (*Cotinus coggygria*, *Helichrysum arenarium*, *Origanum vulgare*, *Pistacia vera*, *Salvia fruticosa*, and *Sideritis congesta*) against 30 clinical *C. acnes* strains was screened. The extracts were examined in vitro for hyaluronidase, collagenase, XO and LOX inhibitions, which are associated with inflammation. According to the calculated MIC_50_ and MIC_90_ values of the extracts and the results of enzyme inhibition in vitro, two extracts (*C. coggygria* and *H. arenarium*) were selected, and nanoemulsion formulations were prepared with these extracts. These two extracts were also examined for their phenolic compounds by LC-MS/MS analysis.

In our first screening, the lowest MIC value (19.5 μg/mL) against the standard strain was found in the *H. arenarium* flower extract. The same extract was also best in terms of its formulation preparation, showing 82.51% XO inhibition. The flowers of the plant known as the “ölmez çiçek” in our country have been utilized as a diuretic and biliary agent due to its rich flavone content [52]. Decoctions prepared from its aerial parts have been traditionally used in the treatment of diabetes, the flowers are used to increase gastric and pancreatic secretion and infusions are prepared from its flower state for biliary ailments [53,54,55]. In a study investigating the effect of methanol extract prepared from the aerial parts containing the leaves and stems of *H. odoratissimum* (L.) Sweet against *C. acnes* (ATCC 6919), the extract displayed a potent antimicrobial activity with an MIC value of 7.81 μg/mL. The extract reduced the level of IL-1α cytokines by 11.08% in *C. acnes*-induced human keratinocytes (HaCaT) at a concentration of 100 μg/mL. The extract inhibited the lipase responsible for sebum degradation and causing inflammation with an IC_50_ value of 157 μg/mL, cycloxygenase II (COX-II) with an IC_50_ value of 22.87 μg/mL and hyaluronidase with an IC_50_ value of 145.45 μg/mL [56]. Although a different *Helichrysum* species was used in the study, high antimicrobial efficacy against the *C. acnes* strain as in our extract and anti-inflammatory activity were detected. In a study conducted in Türkiye, the antimicrobial efficacy of the methanolic extracts prepared from the aerial parts of *H. arenarium* subsp. *erzincanicum*, *H. arenarium* subsp. *rubicundum*, *H. armenium* subsp. *araxinum* and *H. plicatum* subsp. *pseudoplicatum* was investigated against *Aeromonas hydrophila* ATCC 7965, *Bacillus brevis* FMC 3, *B. cereus* RSKK 863, *B. subtilis* ATCC 6633, *Escherichia coli* ATCC 25922, *Klebsiella pneumoniae* FMC 5, *Morganella morganii*, *Mycobacterium smegmatis* RUT, *Proteus mirabilis* BC 3624, *Pseudomonas aeruginosa* ATCC 27853, *Staphylococcus aureus* ATCC 29213 (A), *S. aureus* ATCC 25923 (B), *Yersinia enterocolitica* ATCC 1501, *Candida albicans* ATCC 1223 and *Saccharomyces cerevisiae* BC 5461 strains. When the results were examined, the taxa possessed antimicrobial activity against *A. hydrophila*, *B. brevis*, *B. cereus*, *K. pneumoniae*, *P. aeruginosa* and *S. aureus* (A) strains. In the high-performance liquid chromatography (HPLC) analysis performed in the same study, *p*-coumaric acid, resveratrol and chlorogenic acid, caffeic acid, apigenin, apigenin-7-glucoside, luteolin and naringenin were detected in *H. arenarium* extracts [57]. Similarly, naringenin (14.888 mg/g extract), luteolin (9.487 mg/g extract), apigenin (6.15 mg/g extract), chlorogenic acid (4.481 mg/g extract), caffeic acid (2.054 mg/g extract) and *p*-coumaric acid (0.789 mg/g extract) were detected in our LC-MS/MS analysis on *H. arenarium* extract.

The screening against the standard strains determined that the most active extract after *H. arenarium* was *P. vera* (pistachio) outer-shell extract (39 μg/mL). When the in vitro enzyme inhibition capacity of the same extract was examined, it exhibited low hyaluronidase inhibition, did not inhibit collagenase, and inhibited the XO by nearly 50% at a 2 mg/mL stock concentration. In the literature review, no study investigated the effectiveness of *P. vera* shell extract against *C. acnes*. In previous studies, the antimicrobial activity of *n*-hexane extract prepared from the bark of the plant and the essential oil obtained from the bark of the *P. vera* “Bronte” variety have been investigated. The *n*-hexane extract exhibited little antibacterial activity but high antifungal activity at 128–256 μg/mL concentrations [58]. The study involving the essential oil demonstrated efficacy against *S. aureus* ATCC6538, *E. coli* ATCC10536, *S. aureus* MRSA ATCC43300, *S. aureus* 74CCH, *S. aureus* 7786, and *S. aureus* 815 strains at a concentration of 7.11 mg/mL, except for *P. aeruginosa* ATCC 9027 (Smeriglio et al., 2017; Hurkul, 2021). In another study, extracts prepared from the shells showed bactericidal and bacteriostatic effects against *S. aureus*. It was determined that the most effective extract against methicillin-resistant *S. aureus* strains was aqueous extract (MIC = 3.12 mg/mL) [59]. Research has demonstrated the antimicrobial efficacy of *P. vera* extracts against strains beyond *C. acnes*.

*C. coggygria*, *O. vulgare*, *S. fruticose* and *S. congesta* extracts, whose efficacy against clinical strains was screened, had MIC values against standard strains of 78 μg/mL. *O. vulgare*, *S. fruticosa* and *S. congesta* are the plant species in the Lamiaceae family and are rich in essential oils. Antimicrobial activity studies against different strains have been carried out on the extracts of the species mentioned above and the essential oils obtained from these species [60,61,62,63,64,65,66,67]. In a study investigating the effects of seven essential oils used in folk medicine in the Mediterranean on *C. acnes* and *Staphylococcus epidermidis* to determine the anti-acne potential, the most effective essential oil was obtained from *O. vulgare* (MIC= 0.34 and 0.67 mg/mL). The nanoemulsion prepared with *O. vulgare* essential oil was tested in a mouse model of acne, and the formulation showed superior healing and antimicrobial effects compared to the reference antibiotic [42]. The activity noted in *O. vulgare* essential oil may correlate with the efficacy of essential-oil-rich plants against *C. acnes* in our investigation. In addition to the antimicrobial activity of *C. coggygria* leaf extract, it was another preferred extract in the preparation of formulations because it highly inhibited hyaluronidase (79.75% ± 1.76) and XO (80.30% ± 0.61) and moderately inhibited collagenase (52.52% ± 0.88). The essential oil and the extract of the plant leaves are registered in the International Nomenclature of Cosmetic Ingredients (INCI). The leaves of the plant have been used in Anatolia as a wound healer and antipyretic, as well as for diarrhea and bleeding [52,68,69]. In Russia, there are also ethnopharmacological records indicating the use of *C. coggygria* in treating skin diseases, as well as for wound healing properties and increasing skin elasticity [70,71]. When the antimicrobial activity studies carried out on the species were examined, in a study conducted in our country, the antibacterial effects of ethanol, methanol, aqueous, chloroform, acetone and petroleum ether extracts prepared from the leaves of the plant were determined against *B. subtilis*, *E. coli*, *Enterococcus faecalis*, *P. aeruginosa*, *S. typhimurium*, *S. aureus* and *S. epidermis* strains by the disc diffusion method, and it has been determined that the extracts inhibited the growth of these microorganisms at different rates. It has been observed that water and methanol extracts of the plant are highly effective against *S. aureus*, *S. epidermis* and *E. faecalis* [72]. In another study conducted in our country, the antimicrobial effects of the extracts prepared from *C. coggygria* leaves were investigated, and ethyl acetate extract was reported to have a remarkable antifungal effect against *C. albicans* and a high antibacterial effect against *Proteus mirabilis*. Gallic acid and methyl gallate were isolated as the main compounds from the ethyl acetate extract [73]. In another study investigating the antimicrobial, antioxidant and anti-inflammatory effects of extracts prepared from young shoots of the plant, the acetone extract and the ethyl acetate fraction obtained from this extract significantly inhibited the growth of Gram-positive and Gram-negative bacteria (MIC = 25–200 μg/mL), while the chloroform fraction displayed an inhibitory effect of against *C. albicans* [74]. In another study conducted in Italy, the antibacterial effects of hexane, ethanol and water extracts prepared from 72 plants used for wound healing purposes against microorganisms that cause dental caries were examined by the agar diffusion method. The method used *Streptecoccus mutans*, *S. sobrinus*, *Lactobacillus casei* and *Actinomyces viscosus* as oral pathogenic bacteria. The extracts were all dissolved in water containing 10% DMSO and diluted to final concentrations between 12.5 and 200 mg/mL. The study used triclosan, an antigingivitic agent in toothpaste and mouthwash, at a concentration of 0.3% as a positive control. The MIC values of the extracts were calculated using the standard microdilution method in brain heart infusion medium. Among the extracts screened using dilutions of the extracts in the concentration range of 6.25–100 mg/mL, 22 extracts showed remarkable antibacterial effects. The aqueous extract prepared from *C. coggygria* showed the highest effect, and the antibacterial effect was detected against all bacteria at all concentrations. It has been commented that this effect may be due to the high phenolic content of the extract [75]. In the literature review, although no study investigated the activity of the species against *C. acnes*, its antimicrobial effectiveness against several fungal and bacterial strains has been proven.

Our LC-MS/MS analyses revealed that quinic acid is the most abundant compound among the tested phenolic compounds for *H. arenarium* and *C. coggygria* extracts. Quinic acid is a cyclohexane carboxylic acid with antioxidant, antibacterial, antiviral and anti-inflammatory properties [76]. In a study investigating the effect of quinic acid against the cellular functions of *S. aureus* ATCC 6538, it was found that the compound exerts an antibacterial effect by reducing pH_in_, succinate dehydrogenase activity, the intracellular ATP concentration and DNA synthesis [77]. In another study investigating the antibiofilm effect of *Lonicera japonica* Thunb. flowers, quinic acid was concluded to be the active compound of the extract, which may serve as an effective antibiofilm agent against *P. aeruginosa* and associated infections [78].

In this research, the effect of varying the amount of MCT on the characterization of the formulations was investigated. As shown in Table 8, increasing the amount of MCT resulted in an increased in the droplet size of the formulations (from 135.6 ± 1.0 nm (NE-1) to 227.5 ± 1.6 nm (NE-9)). This phenomenon may be attributed to the increased viscosity of the oil phase due to the higher MCT content. The increased viscosity of the formulations could make it more challenging to achieve NEs with smaller droplet sizes [79]. It was intended to have a high amount of MCT in the formulations, as it was evaluated that plant extracts could be retained in the oil phase at a higher rate. Moreover, MCT is used as a carrier oil [80] due to its stability and low oxidation rate, which helps minimize the formation of free radicals over time, thereby preserving stability [81]. Based on this information, the decision was made to continue with the formulation containing the highest amount of MCT, which is also suitable for droplet size. It was determined that the interaction between the CS/HPMC hydrogel and NEs could increase the droplet size [82]. Therefore, the formulation with the highest MCT content, which has a size of less than 200 nm (NE-7), was selected. Additionally, all formulations prepared with varying MCT amounts have a PDI value lower than 0.3, and they all exhibit a negative zeta potential (ranging from −5.4 ± 0.2 mV to −12.7 ± 1.4 mV). The small PDI value (<0.5) indicates a narrow size distribution [83].

The effect of α-tocopherol on the NEs’ characterizations was also investigated. α-Tocopherol is a potent lipophilic antioxidant, and the topical delivery of α-tocopherol promotes skin protection against UV-induced lipid peroxidation [84]. It was added to the formulations in the range of 50–200 mg. An increase in the amount of α-tocopherol was found to increase the droplet size, which may be attributed to its potential effect on increasing the viscosity of the oil phase. With the use of 100 mg (NE-11) and 200 mg (NE-12) of α-tocopherol in formulations, the droplet size reached 284.8 ± 25.2 nm and 313.2 ± 25.1, respectively (Table 8). When using 50 mg of α-tocopherol (NE-10, 243.1 ± 9.5 nm), the droplet size was also increased compared to the formulation without α-tocopherol (NE-7). Still, this increase was more limited compared to the other two formulations (NE-11 and NE-12). In terms of statistical significance, no difference in droplet size was observed between the formulation containing 50 mg α-tocopherol (NE-10) and the formulation containing 100 mg α-tocopherol (NE-11) (*p* > 0.05). Additionally, the α-tocopherol concentration of less than 0.2% in skin care cosmetics is sufficient to protect lipids against peroxidation [85]. These results were consistent with a study conducted by Teixeira et al. [86]. For these formulations, the zeta potential of NEs was close to each other (ranging from −7.0 ± 0.4 to −8.8 ± 0.8 mV). In a study conducted by Sahafi and colleagues, it was stated that increasing the α-tocopherol ratio from 0% to 40% (w/w) did not significantly change the zeta potential [87]. This indicates that α-tocopherol does not affect the zeta potential of NEs or that this change is insignificant. The PDIs of all samples were below 0.3, indicating that the distributions were homogeneous in size. In further research, it was decided to use the NE-10 content in the formulations to be developed by adding oleic acid. All formulations containing different amounts of oleic acid successfully passed accelerated stability tests. Due to its non-toxic nature and biocompatibility, oleic acid is frequently used in pharmaceutical research [88]. In this study, oleic acid was utilized to achieve a stronger ionic interaction with hydrogel (containing CS) due to its negatively charged nature [89]. When the NEs without oleic acid had a droplet size of 243.1 ± 9.5 nm (NE-10), the droplet size of the formulations containing 25 mg and 50 mg of oleic acid decreased to 227.5 ± 7.0 nm (NE-13) and 220.6 ± 5.2 nm (NE-14), respectively (*p* > 0.05) (Table 8). This can be attributed to the higher electrostatic repulsion effect. In formulations containing higher amounts of oleic acid (100 mg and 200 mg), an increase in the net value of the zeta potential was observed despite an increase in droplet size. This could be attributed to an increase in the viscosity of the oil phase. Although the absolute value of the zeta potential increased, the increase in viscosity had a greater effect, resulting in an increase in the droplet size. Except for NE-16, the PDI value of the formulations is less than 0.3. NE-16 has a PDI value of 0.417 ± 0.022. This result indicates that the excessive amount of oleic acid added to the formulation prevented a homogeneous droplet size distribution. Based on these data, the formulation containing 50 mg of oleic acid (NE-14) was selected for preparing extract-loaded formulations.

Statistically significant differences in droplet size were not observed between blank NEs and extract-loaded NEs (*p* > 0.05) (Table 9). In both extract-loaded NEs, as with blank NEs, a PDI value below 0.3 was found. This indicates that the extract-loaded NEs maintain a homogeneous droplet size distribution. Additionally, the extract-loaded NEs show a zeta potential value close to that of the blank NEs.

Before mixing the extract-loaded NEs with the hydrogel formulation, the NE-14 formulation (blank) was combined with the hydrogel formulation at 1:1, 1:2, 1:3 and 1:4 ratios (NEs: hydrogel, *w*/*w*). Upon the centrifugation of these mixtures, phase separation was not observed at the ratios of 1:3 and 1:4 (*w*/*w*). Therefore, it was decided to prepare NEGs with the highest NE ratio: 1:3 *w*/*w*. Although the mixing of NEs with the hydrogel formulation led to an increase in droplet size, this increase was not significant for both extract-loaded NEGs compared to the extract-loaded NEs (*p* > 0.05) (Table 9). The chitosan layer surrounding the droplets may have also contributed to the increase in droplet size [90]. Additionally, the zeta potential of the NEGs was also changed from negative to positive due to the chitosan coating on the dispersed phases of the oil droplets [82].

In the nanoemulsion system, the morphology of droplets is an important feature. The TEM images (Figure 2) confirmed that both the extract-loaded NE and NEG droplets were spherical and in the nano range. The droplets were evaluated to provide the necessary permeability by passing through even the smallest skin pores due to their spherical structure. When evaluating TEM measurements, smaller sizes were observed compared to my DLS measurements. This is because DLS reflects the hydrodynamic diameter of the formulations, which is usually higher than the value observed in the dried state by TEM [91].

## 5. Conclusions

Considering all these results, 30 medicinal plant extracts were screened for their antimicrobial efficacy against acne pathogen *C. acnes* and their inhibitory effects against enzymes of six active extracts, i.e., LOX, XO and hyaluronidase, associated with inflammation were investigated using in vitro methods. Among these extracts, two extracts (*C. coggygria* and *H. arenarium*) with a high level of inhibition were further selected and loaded into optimum nanoemulgel formulations in different trials, and characterization studies of the formulation were conducted. *C. coggygria* and *H. arenarium* are medicinal plants that have been used in Anatolian folk medicine for various purposes over many years. Future studies are planned to test the safety of the extracts on the human keratinocyte cell line (HaCaT). With the findings obtained herein, a national patent application (application number: 2023/019595) for the aforementioned herbal formulations with anti-acne effects was filled.

## Figures and Tables

**Table 1:** The collection sites, dates, and yields of the extracts obtained from the plants used in the screening.

Species	Family	Plant Part	The Collection Sites and Dates in Türkiye	Yield % (*w*/*w*)
*Alchemilla stricta* Rothm.	Rosaceae	Leaves	Trabzon, May 2019	3.21
*Allium schoenprasum* L.	Amaryllidaceae	Leaves	Trabzon, June 2019	4.74
*Artemisia dracunculus* L.	Asteraceae	Leaves	Konya Medicinal and Endemic Plants Education and Research Farm, June 2020	7.67
*Borago officinalis* L.	Boraginaceae	Leaves	Konya Medicinal and Endemic Plants Education and Research Farm, June 2021	5.27
*Calendula officinalis* L.	Asteraceae	Flowers	Konya Medicinal and Endemic Plants Education and Research Farm, July 2020	9.82
*Camellia sinensis* (L.) Kuntze	Theaceae	Leaves	General Directorate of Tea Enterprises (ÇAYKUR), 2013	13.70
*Cotinus coggygria* Scop.	Anacardiaceae	Leaves	Ankara, November 2019	20.77
*Crataegus monogyna* Jacq.	Rosaceae	Leaves	Konya, May 2019	15.38
*Hedera helix* L.	Araliaceae	Leaves	Konya Medicinal and Endemic Plants Education and Research Farm, May 2021	5.57
*Helichrysum arenarium* (L.) Moench	Asteraceae	Flowers	Artvin, July 2019	15.09
*Hippophae rhamnoides* L.	Elaeagnaceae	Leaves	Bayburt, June 2019	11.06
*Laurus nobilis* L.	Lauraceae	Leaves	Samsun, May 2019	12.07
*Lavandula angustifolia* Mill.	Lamiaceae	Flowers	Konya Medicinal and Endemic Plants Education and Research Farm, May 2020	6.99
*Lippia citriodora* (Palau) Kunth (sin: *Aloysia citriodora* Palau)	Verbenaceae	Leaves	Ordu, June 2020	3.52
*Maclura pomifera* (Raf.) C. K. Schneid.	Moraceae	Leaves	Ankara Atatürk Forest Farm, October 2013	7.44
*Origanum majorana* L.	Lamiaceae	Aerial parts	Konya Medicinal and Endemic Plants Education and Research Farm, June 2019	11.32
*Origanum onites* L.	Lamiaceae	Aerial parts	Konya Medicinal and Endemic Plants Education and Research Farm, June 2019	13.21
*Origanum vulgare* L.	Lamiaceae	Aerial parts	Konya Medicinal and Endemic Plants Education and Research Farm, June 2019	14.53
*Pistacia vera* L.	Anacardiaceae	Outer shell	Gaziantep, 2019	11.20
*Prunus laurocerasus* L.	Rosaceae	Leaves	Konya, May 2021	13.10
*Ruscus aculeatus* L.	Asparagaceae	Leaves	Giresun, June 2021	7.27
*Salvia fruticosa* Mill.	Lamiaceae	Leaves	Konya Medicinal and Endemic Plants Education and Research Farm, June 2019	9.05
*Sambucus nigra* L.	Viburnaceae	Fruits	Konya Medicinal and Endemic Plants Education and Research Farm, June 2020	8.52
*Satureja spicigera* Boiss.	Lamiaceae	Aerial parts	Konya Medicinal and Endemic Plants Education and Research Farm, June 2020	3.62
*Sideritis congesta* P. H. Davis & Hub.-Mor.	Lamiaceae	Flowers	Konya Medicinal and Endemic Plants Education and Research Farm, July 2020	13.90
*Sideritis stricta* Jord. & Fourr.	Lamiaceae	Flowers	Konya Medicinal and Endemic Plants Education and Research Farm, July 2020	17.69
*Thymus nummularius* M. Bieb.	Lamiaceae	Aerial parts	Giresun, June 2020	10.51
*Urtica dioica* L.	Urticaceae	Leaves	Konya Medicinal and Endemic Plants Education and Research Farm, June 2020	2.38
*Vaccinium arctostaphylos* L.	Ericaceae	Leaves	Trabzon, April 2020	20.36
*Vitis vinifera* L.	Vitaceae	Seeds	Denizli, 2019	2.45

**Table 2:** Composition of various blank nanoemulsion formulations.

	Components		
Formulation Code	MCT (mg)	α-Tocopherol (mg)	Oleic Acid (mg)
NE-1	1000	-	-
NE-2	1250	-	-
NE-3	1500	-	-
NE-4	1750	-	-
NE-5	2000	-	-
NE-6	2250	-	-
NE-7	2500	-	-
NE-8	2750	-	-
NE-9	3000	-	-
NE-10	2500	50	-
NE-11	2500	100	-
NE-12	2500	200	-
NE-13	2500	50	25
NE-14	2500	50	50
NE-15	2500	50	100
NE-16	2500	50	200

**Table 3:** The MIC (µg/mL) values of the extracts against the *C. acnes* ATCC 11828 strain.

Species	Plant Parts	MIC (µg/mL)
*Alchemilla stricta*	Leaves	625
*Allium schoenprasum*	Leaves	625
*Artemisia dracunculus*	Leaves	625
*Borago officinalis*	Leaves	625
*Calendula officinalis*	Flowers	312.5
*Camellia sinensis*	Leaves	312.5
*Cotinus coggygria*	Leaves	78
*Crataegus monogyna*	Leaves	>1250 *
*Hedera helix*	Leaves	625
*Helichrysum arenarium*	Flowers	19.5
*Hippophae rhamnoides*	Leaves	625
*Laurus nobilis*	Leaves	625
*Lavandula angustifolia*	Flowers	625
*Lippia citriodora*	Leaves	312.5
*Maclura pomifera*	Leaves	>1250
*Origanum majorana*	Aerial parts	312.5
*Origanum onites*	Aerial parts	312.5
*Origanum vulgare*	Aerial parts	78
*Pistacia vera*	Outer shell	39
*Prunus laurocerasus*	Leaves	625
*Ruscus aculeatus*	Leaves	>1250
*Salvia fruticosa*	Leaves	78
*Sambucus nigra*	Fruits	>1250
*Satureja spicigera*	Aerial parts	625
*Sideritis congesta*	Flowers	78
*Sideritis stricta*	Flowers	156
*Thymus nummularius*	Aerial parts	>1250
*Urtica dioica*	Leaves	625
*Vaccinium arctostaphylos*	Leaves	>1250
*Vitis vinifera*	Seeds	>1250
Reference (Ampicilline)		0.5

* The MIC value exceeds the maximum testable concentration of 1250 µg/mL.

**Table 4:** MIC (µg/mL) values of the extracts against *C. acnes* clinical strains.

Strains		*Cotinus coggygria* (Leaves)	*Helichrysum arenarium* (Flowers)	*Origanum vulgare* (Aerial Parts)	*Pistacia vera* (Shell)	*Salvia fruticosa* (Leaves)	*Sideritis congesta* (Flowers)	AMP
		MIC (µg/mL)						
1	H1	625	156	312.5	78	312.5	625	<0.5
2	H2	1250	156	625	156	625	1250	<0.5
3	H3	1250	39	625	156	625	312.5	<0.5
4	H4	1250	78	625	156	625	625	<0.5
5	H5	1250	156	625	312.5	1250	1250	<0.5
6	H6	625	78	312.5	78	312.5	625	<0.5
7	H7	625	156	312.5	156	625	1250	<0.5
8	H9	1250	78	625	156	625	1250	<0.5
9	H10	1250	78	625	156	625	1250	<0.5
10	H11	625	156	312.5	78	625	1250	<0.5
11	H12	625	156	312.5	156	312.5	625	<0.5
12	H13	625	78	625	156	625	1250	0.5
13	H15	625	156	625	312.5	1250	1250	<0.5
14	H16	1250	78	312.5	78	625	1250	<0.5
15	H17	625	78	625	156	1250	1250	<0.5
16	H18	625	78	625	156	625	1250	<0.5
17	H19	1250	78	312.5	156	625	625	<0.5
18	H21	1250	78	312.5	156	625	625	<0.5
19	H23	1250	156	625	156	1250	1250	0.5
20	K4	625	156	625	156	312.5	1250	0.5
21	K3	625	78	625	78	78	625	0.5
22	K8	625	156	312.5	78	312.5	625	0.5
23	K6	625	156	1250	78	78	1250	0.5
24	K9	625	156	625	156	156	1250	0.5
25	K10	625	156	625	156	156	1250	0.5
26	K11	625	156	625	156	156	1250	0.5
27	K2	625	156	625	78	78	625	0.5
28	K6	625	156	1250	156	78	1250	0.5
29	K5	625	156	625	156	156	625	0.5
30	K7	625	156	625	78	156	625	0.5
31	ATCC 11828	156	312.5	156	156	312.5	312.5	0.5
32	ATCC 11827	625	625	1250	78	312. 5	1250	0.5

H: Strains isolated from patients with active acne, K: Strains isolated from a control group without active acne, American Type Culture Collection (ATCC): Standard strains, AMP: Ampicillin.

**Table 5:** MIC_50_ (µg/mL) and MIC_90_ (µg/mL) values of the extracts against *C. acnes* strains.

Extracts	MIC Range (µg/mL)	MIC_50_ (µg/mL)	MIC_90_ (µg/mL)
*Cotinus coggygria*	156–1250	1250	1250
*Helichrysum arenarium*	39–625	156	156
*Origanum vulgare*	156–1250	625	625
*Pistacia vera*	78–312.5	156	156
*Salvia fruticosa*	78–1250	625	1250
*Sideritis congesta*	312.5–1250	1250	1250

MIC range: the MIC range observed among all the tested clinical *C. acnes* strains; MIC₅₀: the MIC at which 50% of all tested clinical *C. acnes* isolates are inhibited; MIC₉₀: the MIC at which 90% of all tested clinical *C. acnes* isolates are inhibited.

**Table 6:** Hyaluronidase, collagenase, XO and LOX inhibition values of the extracts.

	Hyaluronidase Inhibition (Inhibition% ± S.D. ^a^) 2 mg/mL ^b^	Collagenase Inhibition (Inhibition% ± S.D. ^a^) 2 mg/mL	XO Inhibition (Inhibition% ± S.D. ^a^) 2 mg/mL	LOX Inhibition (Inhibition% ± S.D. ^a^) 2 mg/mL
*C. coggygria*	79.75 ± 1.76	52.52 ± 0.88 ****	80.30 ± 0.61 ****	14.63 ± 0.17 ****
*H. arenarium*	- ^c^	-	82.51 ± 3.81 ****	13.78 ± 2.67 ****
*O. vulgare*	7.68 ± 1.67 ****	11.23 ± 2.51 ****	41.93 ± 2.10 ****	49.37 ± 0.51 ****
*P. vera*	17.24 ± 0.01 ****	-	48.57 ± 2.67 ****	24.44 ± 3.44 ****
*S. fruticosa*	-	-	36.72 ± 2.55 ****	47.57 ± 0.87 ****
*S. congesta*	-	7.88 ± 0.26 ****	19.77 ± 1.90 ****	15.55 ± 1.91 ****
References	77.36 ± 3.34 ^d^	74.94 ± 3.98 ^e^	99.50 ± 0.45 ^f^	89.92 ± 5.51 ^g^

^a^ Standard deviation (n = 3), ^b^ Stock concentration, ^c^ No activity, ^d^ Tannic acid (2 mg/mL), ^e^ 1.10-Phenanthroline (1 mg/mL), ^f^ Allopurinol (1 mg/mL), ^g^ Baicalein (2 mg/mL), **** *p* < 0.0001.

**Table 7:** Identification and quantification of phenolic compounds of *Cotinus coggygria* leaf and *Helichrysum arenarium* aerial part extracts by LC-MS/MS.

No	Analytes	RT ^a^	M.I. (*m*/*z*) ^b^	F.I. (*m*/*z*) ^c^	Ion. Mode	Equation	*r* ^2 d^	*C. coggygria* (mg Analyte/g Extract)	*H. arenarium* (mg Analyte/g Extract)
1	Quinic acid	3.0	190.8	93.0	Neg	*y* = −0.0129989 + 2.97989×	0.996	129.686	69.331
4	Gallic acid	4.4	168.8	79.0	Neg	*y* = 0.0547697 + 20.8152×	0.999	8.048	0.025
6	Protocatechuic acid	6.8	152.8	108.0	Neg	*y* = 0.211373 + 12.8622×	0.957	3.352	0.673
8	Gentisic acid	8.3	152.8	109.0	Neg	*y* = −0.0238983 + 12.1494×	0.997	N.D.	0.212
9	Chlorogenic acid	8.4	353.0	85.0	Neg	*y* = 0.289983 + 36.3926×	0.995	N.D.	4.481
10	Protocatechuic aldehyde	8.5	137.2	92.0	Neg	*y* = 0.257085 + 25.4657×	0.996	0.016	0.089
11	Tannic acid	9.2	182.8	78.0	Neg	*y* = 0.0126307 + 26.9263×	0.999	8.559	N.D.
14	4-OH Benzoic acid	10.5	137,2	65.0	Neg	*y* = −0.0240747 + 5.06492×	0.999	N.D.	0.489
17	Caffeic acid	12.1	179.0	134.0	Neg	*y* = 0.120319 + 95.4610×	0.999	N.D.	2.054
24	*p*-Coumaric acid	17.8	163.0	93.0	Neg	*y* = 0.0249034 + 18.5180×	0.999	0.065	0.789
29	Salicylic acid	21.8	137.2	65.0	Neg	*y* = 0.239287 + 153.659×	0.999	N.D.	0.13
30	Cyranoside	23.7	447.0	284.0	Neg	*y* = 0.280246 + 6.13360×	0.997	0.11	18.93
31	Miquelianin	24.1	477.0	150.9	Neg	*y* = −0.00991585 + 5.50334×	0.999	0.355	N.D.
33	Rutin	25.6	608.9	301.0	Neg	*y* = −0.0771907 + 2.89868×	0.999	0.085	N.D.
34	Isoquercitrin	25.6	463.0	271.0	Neg	*y* = −0.111120 + 4.10546×	0.998	1.444	19.432
35	Hesperidin	25.8	611.2	449.0	Poz	*y* = 0.139055 + 13.2785×	0.999	0.079	N.D.
37	Genistin	26.3	431.0	239.0	Neg	*y* = 1.65808 + 7.57459×	0.991	0.622	52.518
40	Cosmosiin	28.2	431.0	269.0	Neg	*y* = −0.708662 + 8.62498×	0.998	0.457	39.644
41	Quercitrin	29.8	447.0	301.0	Neg	*y* = −0.00153274 + 3.20368×	0.999	6.143	0.013
42	Astragalin	30.4	447.0	255.0	Neg	*y* = 0.00825333 + 3.51189×	0.999	1.569	43.3
43	Nicotiflorin	30.6	592.9	255.0/284.0	Neg	*y* = 0.00499333 + 2.62351×	0.999	0.108	0.037
44	Fisetin	30.6	285.0	163.0	Neg	*y* = 0.0365705 + 8.09472×	0.999	0.013	N.D.
47	Quercetin	35.7	301.0	272.9	Neg	*y* = +0.00597342 + 3.39417×	0.999	0.269	1.19
48	Naringenin	35.9	270.9	119.0	Neg	*y* = −0.00393403 + 14.6424×	0.999	0.053	14.888
50	Luteolin	36.7	284.8	151.0/175.0	Neg	*y* = −0.0541723 + 30.7422×	0.999	0.037	9.487
51	Genistein	36.9	269.0	135.0	Neg	*y* = −0.00507501 + 12.1933×	0.999	N.D.	0.011
52	Kaempferol	37.9	285.0	239.0	Neg	*y* = −0.00459557 + 3.13754×	0.999	0.029	0.272
53	Apigenin	38.2	268.8	151.0/149.0	Neg	*y* = 0.119018 + 34.8730×	0.998	0.006	6.15
55	Chrysin	40.5	252.8	145.0/119.0	Neg	*y* = −0.0777300 + 18.8873×	0.999	0.04	0.015
56	Acacetin	40.7	283.0	239.0	Neg	*y* = −0.559818 + 163.062×	0.997	0.058	3.863

^a^ R.T.: Retention time, ^b^ MI (*m*/*z*): Molecular ions of the standard analytes (*m*/*z* ratio), ^c^ FI (*m*/*z*): Fragment ions, ^d^ *r*^2^: Coefficient of determination, N.D.: Not detected.

**Table 8:** Droplet size, PDI and zeta potential of the formulated NEs (mean ± S.D., n = 3).

Formulation Code	Size (nm)	PDI	Zeta Potential (mV)
NE-1	135.6 ± 1.0	0.126 ± 0.014	−5.6 ± 0.5
NE-2	152.5 ± 1.3	0.127 ± 0.002	−7.0 ± 1.3
NE-3	158.1 ± 5.8	0.121 ± 0.008	−6.9 ± 1.3
NE-4	175.6 ± 3.9	0.109 ± 0.007	−10.4 ± 1.2
NE-5	171.1 ± 1.3	0.129 ± 0.004	−10.0 ± 0.6
NE-6	177.3 ± 3.3	0.145 ± 0.014	−12.7 ± 1.4
NE-7	178.0 ± 3.3	0.179 ± 0.011	−10.0 ± 1.3
NE-8	204.6 ± 2.4	0.195 ± 0.049	−10.7 ± 2.4
NE-9	227.5 ± 1.6	0.245 ± 0.055	−5.4 ± 0.2
NE-10	243.1 ± 9.5	0.223 ± 0.013	−8.8 ± 0.8
NE-11	284.8 ± 25.2	0.276 ± 0.068	−8.1 ± 0.6
NE-12	313.2 ± 25.1	0.238 ± 0.055	−7.0 ± 0.4
NE-13	227.5 ± 7.0	0.159 ± 0.013	−12.6 ± 2.3
NE-14	220.6 ± 5.2	0.195 ± 0.030	−13.3 ± 1.6
NE-15	232.4 ± 5.3	0.202 ± 0.021	−16.4 ± 1.3
NE-16	267.7 ± 27.4	0.417 ± 0.022	−21.6 ± 0.4

**Table 9:** Droplet size, PDI and zeta potential of selected blank NEs and extract-loaded NEs (mean ± S.D., n = 3).

Formulation Code	Size (nm)	PDI	Zeta Potential (mV)
NE-14	220.6 ± 5.2	0.195 ± 0.030	−13.3 ± 1.6
CC-NE	249.1 ± 15.0	0.271 ± 0.031	−11.2 ± 1.2
HA-NE	239.4 ± 13.1	0.261 ± 0.037	−14.7 ± 0.6
CC-NEG	271.1 ± 3.6	0.237 ± 0.016	24.1 ± 4.3
HA-NEG	277.4 ± 25.9	0.245 ± 0.014	26.1 ± 2.9

## Data Availability

The original contributions presented in this study are included in the article. Further inquiries can be directed to the corresponding author.

## Abbreviations

The following abbreviations are used in this manuscript:

ATCC	American Type Culture Collection
BHIB	Brain heart infusion broth
CLSI	Clinical and Laboratory Standards Institute
CS	Chitosan
FALGPA	N-(3-[2-furyl]acryloyl)-Leu-Gly-Pro-Ala
HA	Hyaluronic acid
HPMC	Hydroxypropyl methylcellulose
LOX	Lipoxygenase
MCT	Medium-chain triglyceride
MIC	Minimum inhibitory concentration
MMP	Matrix metalloproteinase
NEGs	Nanoemulgels
NEs	Nanoemulsions
PDI	Polydispersity index
TDDS	Transdermal drug delivery system
TEM	Transmission electron microscopy
UV	Ultraviolet
XD	Xanthine dehydrogenase
XO	Xanthine oxidase
